# Random regression for modeling soybean plant response to irrigation changes using time-series multispectral data

**DOI:** 10.3389/fpls.2023.1201806

**Published:** 2023-07-05

**Authors:** Kengo Sakurai, Yusuke Toda, Kosuke Hamazaki, Yoshihiro Ohmori, Yuji Yamasaki, Hirokazu Takahashi, Hideki Takanashi, Mai Tsuda, Hisashi Tsujimoto, Akito Kaga, Mikio Nakazono, Toru Fujiwara, Hiroyoshi Iwata

**Affiliations:** ^1^ Graduate School of Agricultural and Life Sciences, University of Tokyo, Tokyo, Japan; ^2^ Arid Land Research Center, Tottori University, Tottori, Japan; ^3^ Graduate School of Bioagricultural Sciences, Nagoya University, Nagoya, Japan; ^4^ Faculty of Life and Environmental Sciences, University of Tsukuba, Tsukuba, Japan; ^5^ Tsukuba Plant Innovation Research Center, University of Tsukuba, Tsukuba, Japan; ^6^ Soybean and Field Crop Applied Genomics Research Unit, Institute of Crop Science, National Agriculture and Food Research Organization, Tsukuba, Japan

**Keywords:** plant response, irrigation change, drought stress, single environmental trial, multispectral (MS), time-series, random regression model (RRM), *Glycine max* (L.) Merr.

## Abstract

Plant response to drought is an important yield-related trait under abiotic stress, but the method for measuring and modeling plant responses in a time series has not been fully established. The objective of this study was to develop a method to measure and model plant response to irrigation changes using time-series multispectral (MS) data. We evaluated 178 soybean (*Glycine max* (L.) Merr.) accessions under three irrigation treatments at the Arid Land Research Center, Tottori University, Japan in 2019, 2020 and 2021. The irrigation treatments included W5: watering for 5 d followed by no watering 5 d, W10: watering for 10 d followed by no watering 10 d, D10: no watering for 10 d followed by watering 10 d, and D: no watering. To capture the plant responses to irrigation changes, time-series MS data were collected by unmanned aerial vehicle during the irrigation/non-irrigation switch of each irrigation treatment. We built a random regression model (RRM) for each of combination of treatment by year using the time-series MS data. To test the accuracy of the information captured by RRM, we evaluated the coefficient of variation (CV) of fresh shoot weight of all accessions under a total of nine different drought conditions as an indicator of plant’s stability under drought stresses. We built a genomic prediction model ( ${\text{MT}}_{\text{RRM}}\;\text{model}$ ) using the genetic random regression coefficients of RRM as secondary traits and evaluated the accuracy of each model for predicting CV. In 2020 and 2021,the mean prediction accuracies of ${\text{MT}}_{\text{RRM}}\;\text{models}$ built in the changing irrigation treatments (*r* = 0.44 and 0.49, respectively) were higher than that in the continuous drought treatment (*r* = 0.34 and 0.44, respectively) in the same year. When the CV was predicted using the ${\text{MT}}_{\text{RRM}}\;\text{model}$ across 2020 and 2021 in the changing irrigation treatment, the mean prediction accuracy (*r* = 0.46) was 42% higher than that of the simple genomic prediction model (*r* =0.32). The results suggest that this RRM method using the time-series MS data can effectively capture the genetic variation of plant response to drought.

## 1 Introduction

Soybean (*Glycine max* (L.) Merr.) exhibits a 40% reduction in yield due to drought (Specht et al., 1999), and genetic improvement of drought tolerance in soybeans is needed. Plant responses to drought stress are associated with drought tolerance (Pathan et al., 2014; Ye et al., 2020), and phenotypic data can be collected non-destructively from plants using high-throughput phenotyping (HTP). The relationship between spectral reflectance collected by hyperspectral (HS) and multispectral (MS) cameras and drought stress has been reported in several studies (Winterhalter et al., 2011; Bi et al., 2018; Wijewardana et al., 2019). Relationships between the normalized difference vegetation index (NDVI), which is calculated from the reflectance of near-infrared and red spectra, and the level of wilting have been reported in soybeans (Zhou et al., 2020). In soybeans, genotypes with slow-wilting traits exhibit high yield under drought conditions (Du et al., 2009; Pathan et al., 2014). Among several vegetation indices (VIs), normalized difference red-edge (NDRE), calculated from the reflectance of red-edge and red spectra, is reported to be the best vegetation index for detecting drought stress (Zygielbaum et al., 2009). These plant responses to irrigation changes are continuous, and time-series data can be collected using HTP (Yang et al., 2017). New insights can be obtained by capturing and analyzing time-series plant responses to irrigation changes, which are collected using HTP (Moreira et al., 2020). Chen et al. collected time-series changes in the digital volume of barley calculated from plant images collected using HTP during drought and recovery periods. The speed of recovery differs among genotypes; the faster the speed of recovery, the larger the final biomass (Chen et al., 2014).

Because the shape of time-series changes of biomass, leaf area index, and plant height was an S-shaped curve, they were commonly modeled using a logistic function (Setiyono et al., 2008; Sun and Frelich, 2011; Paine et al., 2012). When the shape of the curve or the function describing the curve is not obvious, smooth functions, such as Legendre polynomials or spline functions, can be used to model time series changes (Yang et al., 2006; van Eeuwijk et al., 2019). The random regression model (RRM) has been widely used for genetic analysis of time-series data (Campbell et al., 2018; Oliveira et al., 2019; Freitas Moreira et al., 2021). In the RRM, the covariance between each time point in a multivariate mixed model is modeled using Legendre polynomials and spline functions based on the assumption that time-series data are changing continuously (Henderson, 1984). The RRM makes it possible to describe time-series random genetic effects using a small number of parameters, i.e., regression coefficients (Huisman et al., 2002; Schaeffer, 2004). Estimated random regression coefficients can be used in a genome-wide association study (GWAS) to search for new genes (Campbell et al., 2019) or as secondary traits in a multi-trait model (MTM) to increase the prediction accuracy of the target trait (Sun et al., 2017).

With respect to drought tolerance, one of the important breeding targets is “stability under drought stress”, which is a phenotype that is stable under different drought levels (i.e., severe, moderate, and mild) caused by different rainfall and water availability in different years and locations (Pidgeon et al., 2006; Kumar et al., 2012; Kumar et al., 2018; Torres and Henry, 2018; Ayed et al., 2021). Coefficient of variation (CV) of phenotypes in a target trait calculated from multi-environmental trials has long been used as an indicator for the stability over environments (Rao and Willey, 1980; Mohammadi and Amri, 2008; Das et al., 2010; Küchenmeister et al., 2012; Ray et al., 2015; Di Matteo et al., 2016). ¨ CV is calculated by dividing the standard deviation of phenotypes of the target trait by its mean, with smaller values indicating greater stability (Francis and Kannenberg, 1978). To evaluate the under-drought-stress stability with CV of the trait, field trials must be conducted at various drought levels. Conducting multi-environmental trials and evaluating the CVs for new genotypes is, however, time-consuming and costly. Predicting CV based on plant responses to irrigation changes may greatly reduce the time and cost of the CV evaluation. Although the relationship between the plant responses to irrigation changes and drought tolerance has been reported (Chen et al., 2014; Marchetti et al., 2019; Dodig et al., 2021), no studies have modeled plant response to irrigation changes and, based on the model, evaluated its relationship with plant’s stability under drought stress.

The objective of this study was to develop a method to measure and model plant responses to changes in irrigation. The developed method was applied to a single environmental trial with multiple irrigation patterns, and its effectiveness was evaluated based on its relationship to plant’s stability under drought stress. In this study, we evaluated 178 soybean accessions under different irrigation treatments in a 3-year trial. CV was calculated using fresh shoot weights observed in nine combinations of treatments by years and was used as an indicator of the plant’s stability under drought stress. Time-series MS data for each year were modeled using RRMs, and the calculated genetic random regression coefficients were used as secondary traits for the genomic prediction of CV. If genomic prediction models using the calculated genetic random regression coefficients as secondary traits show higher prediction accuracy than those of the simple genomic prediction model without secondary traits, this suggests that time-series MS data are useful in predicting CV. If the genomic prediction models using the time-series MS data collected in the changing irrigation treatments show higher prediction accuracy than those in the continuous drought treatment, this suggests that time-series changes in MS data caused by irrigation change are useful in evaluating plant’s stability under drought stress. Additionally, we built three different prediction models: (1) within each combination of treatments by years, (2) using a small dataset of secondary traits, and (3) across years for the same type of treatment. Prediction model 1 could be used to predict the CV of novel genotypes within the same year. Prediction model 2 aimed to reduce the amount of data with secondary traits in the training set and the cost of collecting secondary traits. Prediction model 3 was intended to predict the CV of novel genotypes over the years using a previously prepared prediction model. Based on these three cases, we evaluated the effectiveness of the method for modeling time-series MS data using RRM.

## 2 Materials and methods

### 2.1 Experimental data

**Figure 1:**
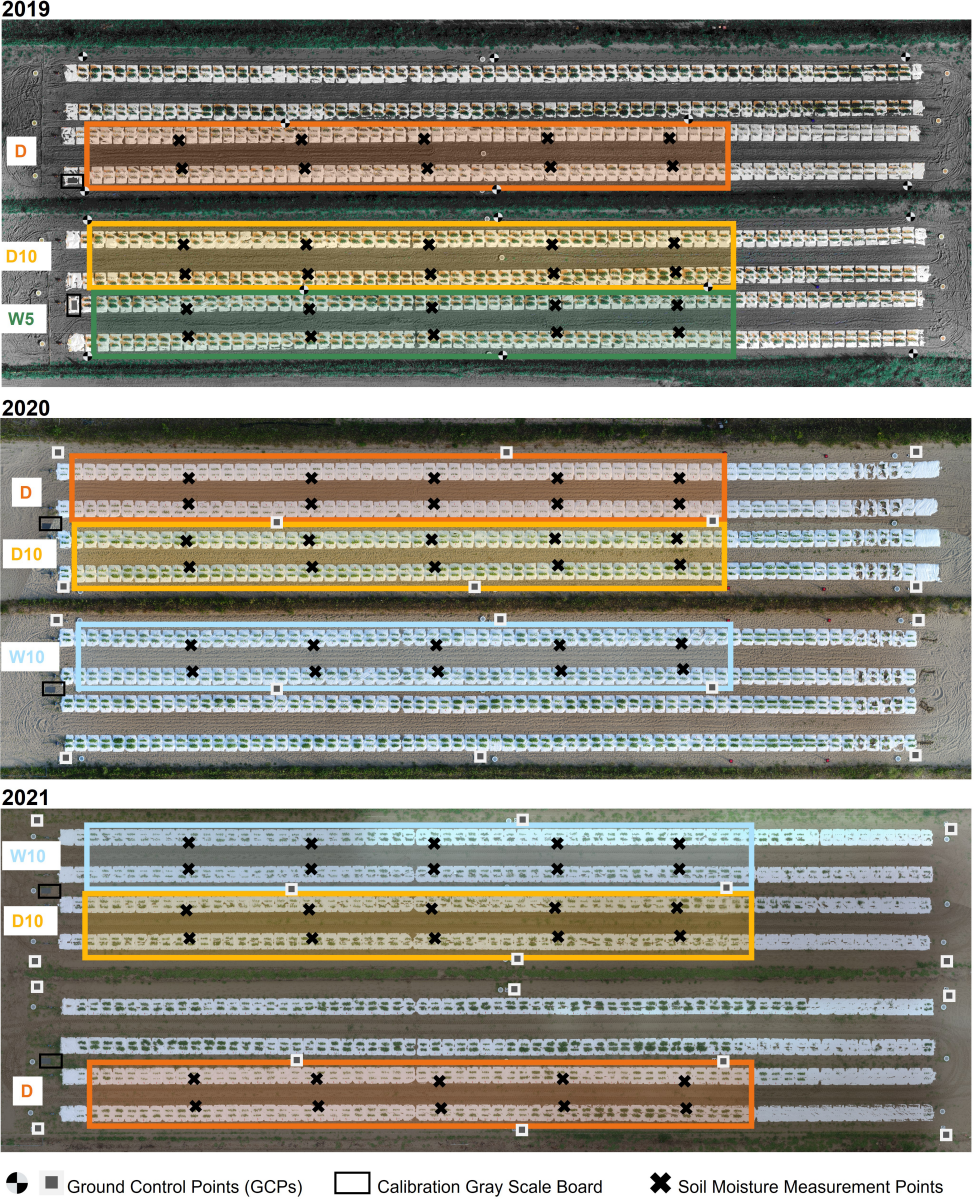
Experimental field overview and treatments in each year. W5: watering for 5 d followed by no watering for 5 d, W10: watering for 10 d followed by no watering for 10 d, D10: no watering for 10 d followed by watering for 10 d, D: no watering treatment.

**Figure 2:**
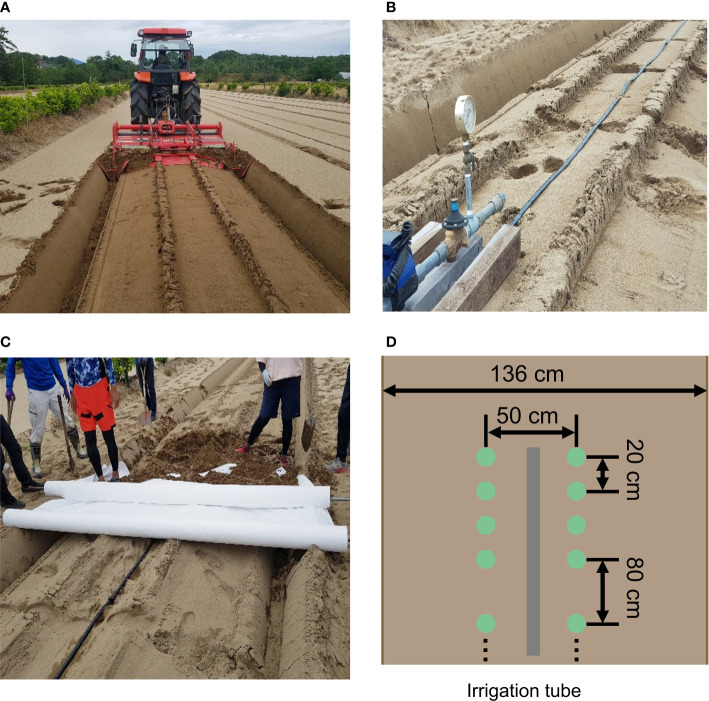
The setup of the experimental field. **(A)** Ridge for each treatment. **(B)** Irrigation tube on each ridge. **(C)** White multi-sheet. **(D)** Planting pattern in each ridge. Pictures **(A–C)** were used in Bui et al. (Bui et al., 2022). Permission to use these pictures was granted by the author.

In this study, the accessions and experimental fields were the same as in the previous study (Sakurai et al., 2022). The diverse panel of 178 soybean accessions used were obtained from the gene bank of the National Institute of Agrobiological Sciences, Tsukuba, Japan (**Table S1**). These 178 accessions mainly consisted of Japanese and global soybean minicore collections (Kaga et al., 2011; Kajiya-Kanegae et al., 2021). A total of 178 soybean accessions were grown in three years 2019, 2020, and 2021 in the same experimental field at the Arid Land Research Center, Tottori University, Japan (35°32’ N lat, 134°12’ E long, 14 m above sea level). The experimental field soil was sandy and retained high water permeability. We used four types of drought treatments: no watering (drought, Treatment D), watering for 5 d followed by no watering for 5 d (Treatment W5), watering for 10 d followed by no watering for 10 d (Treatment W10), and no watering for 10 d followed by watering for 10 d (Treatment D10). Treatments D, W5, and D10 were implemented in 2019. Treatments D, W10, and D10 were implemented in 2020 and 2021 (**Figure 1**). Each treatment consisted of two ridges. Each ridge consisted of two rows and one irrigation tube, and microplots were placed parallel to each other on either side of the tubes. The ridge height and width were 30 and 136 cm, respectively. For each treatment, 178 accessions were randomly assigned to microplots per year. As each accession was assigned one microplot per treatment, there were no replicates per treatment. The reason for not taking replications but prioritizing the number of genotypes is that previous studies have shown that genomic predictive modeling and QTL analysis are better with a higher number of genotypes, even if the number of replications is set to one (Knapp and Bridges, 1990; Lorenz, 2013). Four plants of each accession were grown in each microplot. The distances between the rows, microplots, and plants were 50, 80, and 20 cm, respectively (**Figure 2**). Fertilizers (13, 6.0, 20, 11, 7.0 g/m2 115 of N, P, K, Mg, and Ca, respectively) were applied to the field prior to sowing. Sowing was performed on 10 July 2019, 8 July 2020, and 6 July 2021. Two to three seeds were sown at each position, after which the germinated seedlings were thinned to one per position 2 weeks after sowing.

White mulch sheets (Tyvec; DuPont, Wilmington, USA) were laid over the ridges to prevent rainwater infiltration into the soil and control soil drought levels (**Figure 1**). A watering tube was installed under the mulch sheets at the center of each row. The watering tube (JKC Agro, Kumamoto, Japan) irrigated at a flow rate of 1.1 L/h m. Watering was done for over 5 h daily (7:00–9:00, 12:00–14:00, and 16:00–17:00), starting the day after seedling thinning for Treatments W5, W10, and D10. The irrigation cycle for each treatment is shown in **Figure S1**. Soil moisture was measured using a soil moisture meter (TDR-341F; Fujiwara Seisakusho, Tokyo, Japan) at 10 sites for each treatment over 30 d. Except for rainy days, no more than 3 d were allowed between soil moisture measurements. Soil moisture in each treatment was 127 calculated as the average of the treatments in the year (**Figure S1**). Days to flowering (DTF), which was defined as the date that 50% of plants flowered in each microplot (**Figure S2**), were determined. To record DTF, we visited each microplot every other day during the flowering period. In each microplot of the four plants, two centrally located plants were collected 62 d after sowing, and the fresh aboveground weight of each plant was measured. The phenotypic value of the fresh weight of each microplot was calculated as the average of the two plants measured for each microplot. The number of rainy days was 19, 12, and 22 during the field experiment in 2019, 2020, and 2021, respectively.

### 2.2 Multispectral data collection and processing

The MS image collection and image analysis referred to the method employed by Sakurai et al. (Sakurai et al., 2022). In each treatment of a year, MS images were collected using unmanned aerial vehicles (UAVs). In 2019 and 2020, MS images (1.0 cm/pixel) were collected using a four-eye MS camera (Xacti, Osaka, Japan) mounted on a quadcopter UAV (DJI Matrice 100; DJI, Shenzhen, China). The MS camera has four independent lenses and sensors attached to different filters (MidOpt, Palatine, USA), including a triple-bandpass filter (TB550/660/850) and a red-edge bandpass. The TB550/660/850 can collect spectral intensities at 550 nm (green), 660 nm (red), and 850 nm (near-infrared). Bi725 can collect the spectral intensity at 725 nm (red-edge). The MS camera was set for continuous data capture at two frames per second per lens for a total of eight frames per second. The overlap and sidelap rates were set to 90% and the flights were set to an altitude of 20 m. In 2021, MS images (0.74 cm/pixel) were collected using DJI Phantom 4 Multispectral (P4M; DJI, Shenzhen, China). P4M has one RGB sensor and five spectral-band sensors at 450 nm (blue), 560 nm (green), 650 nm (red), 730 nm (red-edge), and 840 nm (near-infrared). P4M continuously collected MS images every two seconds during each flight. The overlap and sidelap rates were set to 75% and the flights were set to an altitude of 15 m.

**Figure 3:**
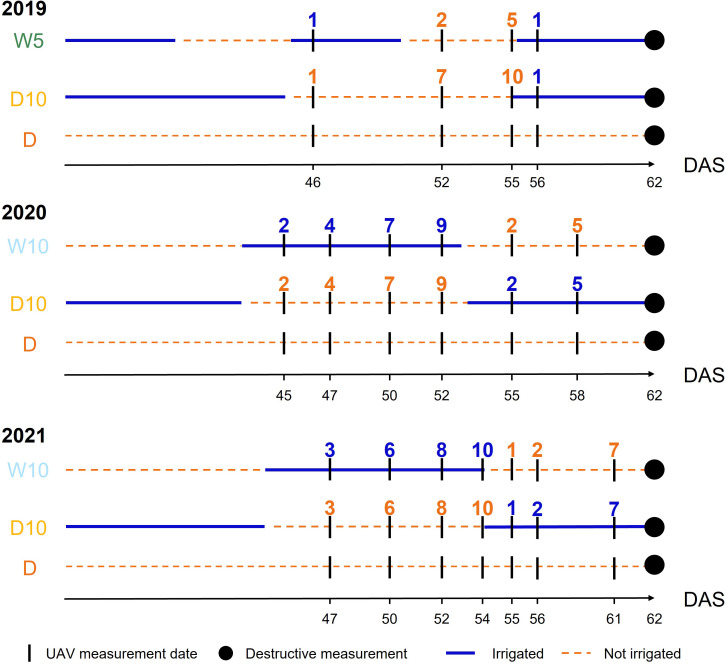
The timings of unmanned aerial vehicle measurements in each year. Blue (respectively Orange) numbers indicate the number of days elapsed since the start of irrigation (respectively drought) treatment. DAS: days after sowing, W5: watering for 5 d followed by no watering 5 d, W10: watering for 10 d followed by no watering 10 d, D10: no watering for 10 d followed by watering 10 d, D: no watering treatment.

Each spectral reflectance was calculated as the ratio of each spectral intensity from a grey scale panel set in the experimental field (**Figure 1**). All the flights were scheduled for 11:00-13:00 under clear sky conditions. Images were collected four, six, and seven times in 2019, 2020, and 2021, respectively (**Figure 3**). UAVs measurements were scheduled before and after the irrigation treatment was switched on W10 and D10 to capture the response of plants to changes in irrigation. Orthomosaic images of each spectral reflectance were obtained using a Pix4Dmapper (Pix4D, Prilly, Switzerland). Sixteen ground control points (GCPs) were set up in the field annually. The positions of the GCPs were measured using Aeropoints (Propeller, Sydney, Australia). Using geolocation information from the GCPs, 792 microplots, each with a maximum of four plants, were segmented from the orthomosaic images. Plants were segmented from an image of each microplot using the NDVI-based segmentation method to extract and analyze the MS image data from only the plants. In this experimental field, it was reported that the NDVI differed significantly from the soil surface, white mulch sheets, and plants in 2019 (Sakurai et al., 2022); thus, plants were segmented with the NDVI threshold set to 0.15, as applied by Sakurai et al. (Sakurai et al., 2022). These analyses (extraction of spectral reflectance, calculation of NDVI, and NDVI-based segmentation) were performed using the OpenCV v3.3.1, library in Python v3.6.8.

Based on the spectral reflectance values of each plant pixel segmented in each microplot, we calculated two types of VIs: NDVI (Price and Bausch, 1995; Zarate-Valdez et al., 2015) and NDRE (Jorge et al., 2019). The equations for these VIs are listed in **Supplementary Table S2**. As there were four plants in each microplot, MS data were collected as a community of four plants within each microplot without considering the overlap between plants. Although we attempted to segment only the plant pixels, background noise was not completely removed. In each microplot, the average VI value may have been heavily influenced by the background noise. To reduce this effect, the median of all the segmented plant pixels was used as the representative value of each microplot. The VIs were calculated using R v4.1.2.

### 2.3 Genotyping

The genome dataset was the same as that used by Sakurai et al. (Sakurai et al., 2022). Whole-genome sequence data were obtained for all accessions (Kajiya-Kanegae et al., 2021). All accessions were genotyped using an Illumina HiSeq X Ten or HiSeq 4000 (Illumina, San Diego, USA), and 4,776,813 single-nucleotide polymorphisms (SNPs) were identified. Among these SNPs, those that were heterozygous or those in which >95% of the individuals had missing data were excluded. Markers were also filtered for a minor allele frequency<0.025, and missing data were imputed based on the mean, after which they were filtered again for a minor allele frequency<0.05. Finally, linkage disequilibrium was computed only for SNP pairs for which the distance was<25,000 base pairs, and SNPs with linkage disequilibrium below 0.8 were selected, resulting in 173,583 SNP markers. Using these SNP markers, the additive numerator relationship matrix G was estimated using the ‘calcGRM’ function in the ‘RAINBOWR’ package in R v0.1.25 (Hamazaki and Iwata, 2020).

### 2.4 Random regression model

The MS data were collected at four, six, and seven time points in 2019, 2020, and 2021, respectively. The ratio of flowering at the timings of UAV measurements in each combination of treatments was calculated using DTF data (**Table S3**). A time series of VI values was modeled using an RRM (Mrode, 2014) for each treatment of each year separately. The RRM takes the following form:


(1) $$y_{it}=b_{t}+\sum_{k=0}^{nr}\phi_{k}\left(t\right)u_{ik}+e_{it}$$

where $y_{it}$ is the phenotypic value of each VI (NDVI or NDRE) at the time point t (t=1,…,4 in 2019, t=1,…,6 in 2020, t=1,…,7 in 2021) for genotype i (i=1,…,178) , $b_{t}$ is the fixed effect of each time point, nr is the order of Legendre polynomial for the genetic effects, $\phi_{k}\left(t\right)$ is the k th (k=0,…,nr) Legendre polynomials for time point t , $u_{ik}$ is the genetic effect for the k th coefficients of Legendre polynomials, and $e_{it}$ is the random residual effect. Vector ${\text{u}}_{k}={\left(u_{1k},\ldots ,u_{178k}\right)}^{T},\text{u}={\left({\text{u}}_{0}^{T},\ldots ,{\text{u}}_{nr}^{T}\right)}^{T}$ follows the multivariate normal (MVN) distribution, $\text{u}∼\text{MVN}\left(0,\text{Q}⊗{\text{I}}_{178}\right)$ where Q is (nr+1)×(nr+1) (co)variance matrix for the Legendre polynomials and ${\text{I}}_{178}$ is 178×178 identical matrix. Vector ${\text{e}}_{k}={\left(e_{1k},\ldots ,e_{178k}\right)}^{T},\text{e}={\left({\text{e}}_{0}^{T},\ldots ,{\text{e}}_{nr}^{T}\right)}^{T}$ follows the MVN distribution $\text{e}∼\text{MVN}\left(0,{\text{I}}_{178\left(\text{nr}+1\right)}\sigma_{e}^{2}\right)$ , where ${\text{I}}_{178\left(nr+1\right)}$ is the identical matrix and $\sigma_{e}^{2}$ is the residual variance. To determine the order of nr , we built RRMs with three values of nr (nr=0,1,2) using the data collected in 2021, because the number of time points was the largest in 2021 among other years. The goodness of the model fit was assessed by computing Akaike’s information criterion (AIC) (Akaike, 1974). The best value of nr is selected based on the lowest AIC value. This RRM model was built using the “ASREML” R package v4.1.0.154 (Gilmour et al., 2015).

### 2.5 Coefficient of variation

We used CV (Francis and Kannenberg, 1978) of fresh weight as an indicator of plant’s stability under drought stresses. CV was calculated as follows:


(2) $$CV_{i}=\frac{\sigma_{i}}{\mu_{i}}\times 100$$

where $\mu_{i}$ is the mean fresh weight in all nine combinations of treatments by years for genotype i (i=1,…,178) , $\sigma_{i}$ is the standard deviation of the fresh weight in the combinations. A low CV value indicates high environmental stability. Before calculating $\mu_{i}$ and $\sigma_{i}$ , fresh weight was scaled by min-max normalization, ranging from 0 to 1 for each combination of treatments by years. This is because $CV_{i}$ is significantly affected by specific combinations of treatments by years, which have large mean values.

### 2.6 Genomic heritability and genomic prediction

Simple genomic prediction models were built to calculate the genomic heritability (Litchfield et al., 2015) for three traits; fresh weight in each combination of treatments by years, the genetic random regression coefficient of RRMs, and CV. Also, a simple genomic prediction model was built to predict the genetic value of the CV. The simple genomic prediction model has the following form:


(3) $$y_{i}=\mu +u_{i}+e_{i}$$

where $y_{i}$ is the phenotypic value for each trait of genotype i (i=1,…,178) , μ is the overall mean, $u_{i}$ is the genetic random effect, and $e_{i}$ is the residual random effect. The vector $\text{u}={\left(u_{1},\ldots ,u_{178}\right)}^{T}$ follows the MVN distribution, $\text{u}∼\text{MVN}\left(0,\text{G}\sigma_{g}^{2}\right)$ where G is the additive numerator relationship matrix, and $\sigma_{g}^{2}$ is the additive genetic variance. Vector $\text{e}={\left(e_{1},\ldots ,e_{178}\right)}^{T}$ follows the MVN distribution $\text{e}∼\text{MVN}\left(0,{\text{I}}_{178}\sigma_{e}^{2}\right)$ , where ${\text{I}}_{178}$ is the identical matrix and $\sigma_{e}^{2}$ is the residual variance. Based on the estimated parameters of genetic and residual variances, genomic heritability was calculated as $h^{2}=\frac{\sigma_{g}^{2}}{\sigma_{g}^{2}+\sigma_{e}^{2}}$ . The model was implemented using the ‘EMM.cpp’ function in the ‘RAINBOWR’ package in R v0.1.25 (Hamazaki and Iwata, 2020).

### 2.7 Multitrait model

For each combination of treatments by years, the MTM was built as a Bayesian multivariate Gaussian model (Montesinos-López et al., 2016; Lado et al., 2018) to predict the CV of a genotype. An MTM using M secondary traits takes the following form:


(4) $$y_{im}=\mu_{m}+u_{im}+e_{im}$$

where $y_{i0}$ is the phenotypic value of the CV for genotype i (i=1,…,178) ; $y_{im}\;\left(m=1,\ldots ,M\right)$ is the phenotypic value of the secondary traits; $\mu_{m}$ is the overall mean for trait m (m=0,…,M) ; $u_{im}$ is the genetic random effect (m=0,…,M) ; and $e_{im}$ is the residual random effect (m=0,…,M) . Vector ${\text{u}}_{m}={\left(u_{1m},\ldots ,u_{178m}\right)}^{T},\text{u}={\left({\text{u}}_{0}^{T},\ldots ,{\text{u}}_{M}^{T}\right)}^{T}$ follows the MVN distribution, u∼MVN(0,Σ⊗G) where Σ is (M+1)×(M+1) genetic (co)variance matrix across traits and G is the additive numerator relationship matrix. Vector ${\text{e}}_{m}={\left(e_{1m},\ldots ,e_{178m}\right)}^{T},\text{e}={\left({\text{e}}_{0}^{T},\ldots ,{\text{e}}_{M}^{T}\right)}^{T}$ follows the MVN distribution $\text{e}∼\text{MVN}\left(0,\text{R}⊗{\text{I}}_{178}\right)$ , where R is (M+1)×(M+1) residual (co)variance matrix across traits and ${\text{I}}_{178}$ is the identical matrix.

We built two different types of MTMs: (1) an MTM directly using the VI value at each time point as a secondary trait ( ${\text{MT}}_{\text{All}}\;\text{model}$ ), and (2) an MTM using the genetic random regression coefficients of RRM (Equation 1) as a secondary trait ( ${\text{MT}}_{\text{RRM}}\;\text{model}$ ).

### 2.8 Cross-validation cases

**Figure 4:**
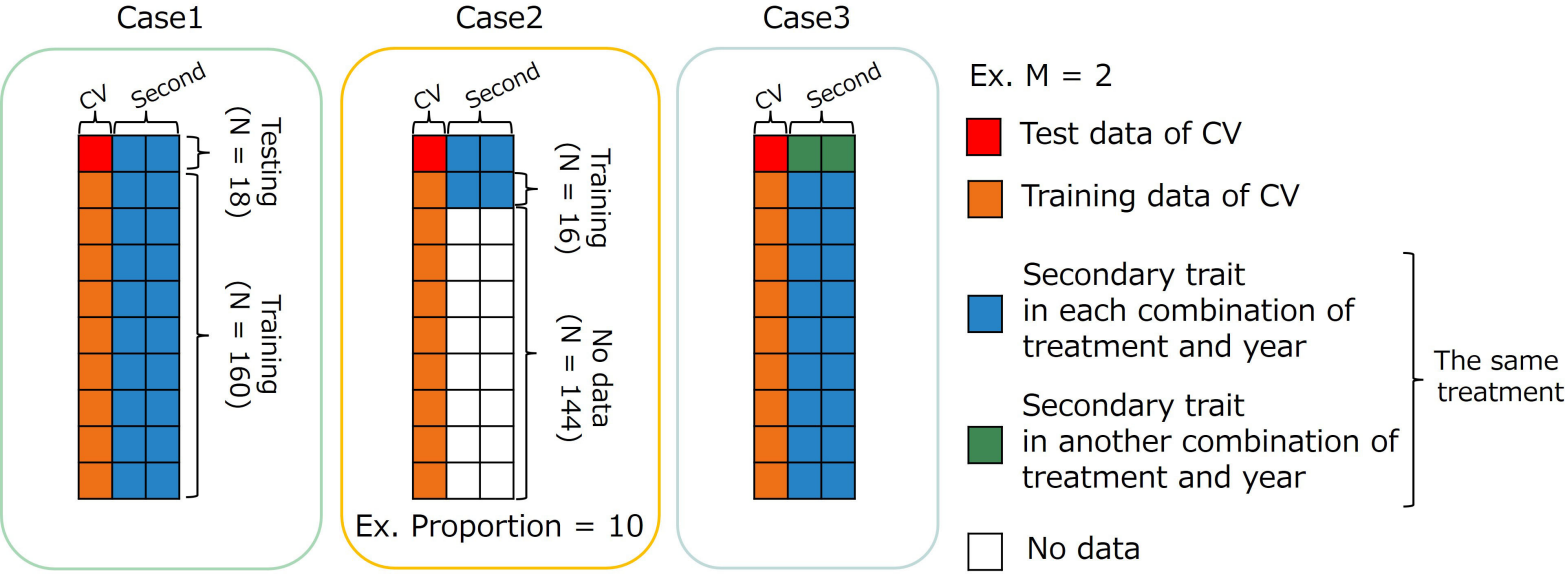
Image representation of three cases using multi-trait (MT) model. The dataset has 178 accessions (N=178). Case1: MT prediction within each combination of treatments by years. Case2: MT prediction within combination of treatments by years with small data set of secondary traits. Case3: MT prediction across years for the same type of treatment. CV, coefficient of variation; M, the number of secondary traits.

We assumed three cases using MTM (**Figure 4**) and compared the prediction accuracies of ${\text{MT}}_{\text{All}}\;\text{model}$ and ${\text{MT}}_{\text{RRM}}\;\text{model}$ . The prediction accuracy was evaluated using a 10-fold cross-validation with 10 replicates. Pearson’s correlations were calculated between the observed and predicted CV values in each replicate, and the average of these correlations was used as the prediction accuracy.

#### 2.8.1 Case 1: within each combination of treatments by years

In the first case (Case1), the whole data set in each combination of treatments by years was divided into training and test sets. Predicting the CV of the test set using secondary traits and genome-wide marker data. Because an MTM was built in each combination of treatments by years in Case1, the number of secondary traits in an MTM need not be equal among years. In ${\text{MT}}_{\text{All}}\;\text{model}$ , M (the number of secondary traits) was four, six, and seven in 2019, 2020, and 2021, respectively. In ${\text{MT}}_{\text{RRM}}\;\text{model}$ , M was equal to nr+1 (the order in Equation 1), which was the same for all years.

#### 2.8.2 Case 2: using a small dataset of secondary traits

In the second case (Case2), we assumed a situation that CV had already been calculated from past data. In MTM, secondary trait data are usually collected for all genotypes in the training and test sets. We have to conduct the field experiment and collect time-series MS data to build an MTM in our study. The small number of genotypes measured for secondary traits in the training set resulted in cost reduction. The proportions of data with secondary traits were set to 10%, 25%, and 50%. First, the whole data set for each combination of treatments by years was divided into training and test sets. Second, genotypes with secondary trait data in the training data were randomly selected five times for each proportion to reduce the effect of specific datasets with secondary traits. Therefore, the prediction accuracy was evaluated via a 10-fold cross-validation with 50 replicates. Because an MTM was built in each combination of treatments by years in Case2, the number of secondary traits in an MTM need not be equal among years. In ${\text{MT}}_{\text{All}}\;\text{model}$ , M was four, six, and seven in 2019, 2020, and 2021, respectively. In ${\text{MT}}_{\text{RRM}}\;\text{model}$ , M was equal to nr+1 for all the years.

#### 2.8.3 Case 3: across years for the same type of treatment

The third case (Case3) was intended to create a prediction model using specific year data and predict CV using data from another year. In this case, we predicted the CV of novel genotypes over the years with their secondary trait data and genome-wide marker data using a previously prepared prediction model built using another year’s data. The prediction accuracy was calculated via cross-year cross-validation using the same treatment. Because Treatment W5 did not have yearly replications, this validation was performed only for treatments W10, D10, and D.

In ${\text{MT}}_{\text{All}}\;\text{model}$ , M was only three because the measurement timings differ among the years. We set the start of irrigation (and drought) in the irrigation changing treatments as a reference point and considered the time difference within a day as the same measurement timing. Therefore, MS data on the dates after sowing (DAS) of 52, 55, and 56 in 2019; 50, 52, and 55 in 2020; and 52, 54, and 55 in 2021 were used in ${\text{MT}}_{\text{All}}\;\text{model}$ (**Figure 3**). However, in ${\text{MT}}_{\text{RRM}}\;\text{model}$ , all time point data were available for modeling because time-series MS data were modeled with nr+1 orders, and the genetic random regression coefficients were used as secondary trait data. In ${\text{MT}}_{\text{RRM}}\;\text{model}$ , M can be fixed at nr+1 for all years.

In all three cases, to compare the prediction accuracy between ${\text{MT}}_{\text{All}}$ and ${\text{MT}}_{\text{RRM}}\;\text{models}$ , we calculated the proportion of improvement. The proportion of improvement is defined as follows:


(5) $$POV\left(\%\right)=\left(\frac{PA_{RRM}-PA_{All}}{PA_{All}}\right)\times 100$$

where POV denotes the proportion of improvement, $PA_{All}$ denotes the prediction accuracy of ${\text{MT}}_{\text{All}}\;\text{model}$ , and $PA_{RRM}$ denotes the prediction accuracy of ${\text{MT}}_{\text{RRM}}\;\text{model}$ .

## 3 Results

### 3.1 Relationship between coefficient of variation and fresh weight

**Figure 5:**
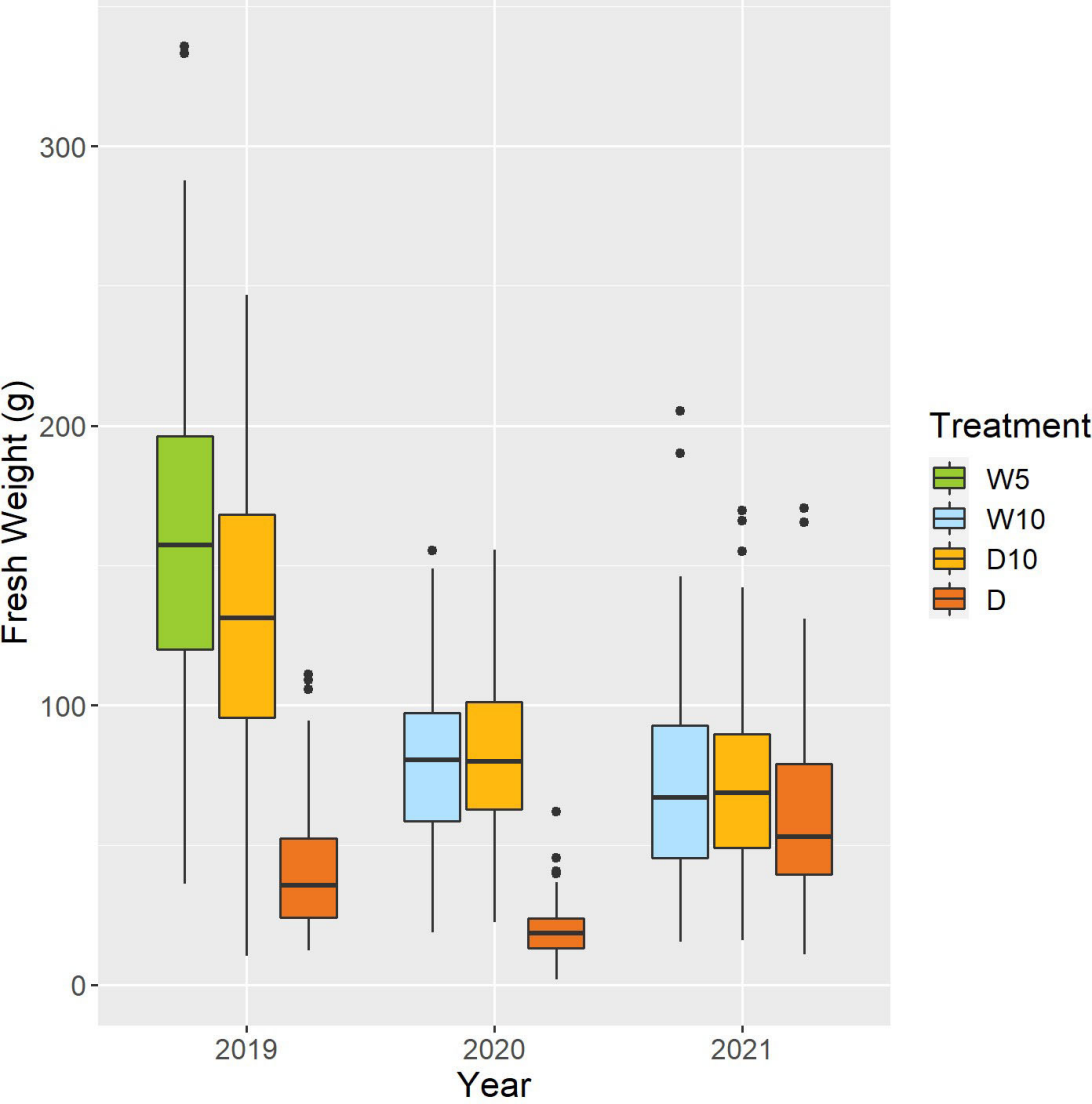
Boxplot of observed fresh weights of 178 soybean accessions in each combination of treatments by years. W5: watering for 5 d followed by no watering 5 d, W10: watering for 10 d followed by no watering 10 d, D10: no watering for 10 d followed by watering 10 d, D: no watering treatment.

Fresh weight varied among the nine combinations of treatments by years (**Figure 5**). As the fresh weight in Treatment D was smaller than that in the other treatments each year, Treatment D was the treatment with the most severe drought stress. For Treatment D10, the fresh weight in 2019 was higher than that in the other two years. Genomic heritability of fresh weight ranged from 0.18 to 0.64 and that of CV was 0.35 (**Table 1**). Next, we calculated the phenotypic correlation between CV and fresh weight for each combination of treatments by years. No positive phenotypic correlation was observed between the CV and fresh weight. In addition, the phenotypic correlation between CV and fresh weight was negative in seven out of nine combinations of treatments by years. This result indicates a positive relationship between fresh weight and the stability under drought stress as assessed by CV and in the seven combinations of treatments and years.

**Table 1:** Genomic heritability of fresh weight in each combination of treatments by years, coefficient of variation (CV) calculated over the nine combinations, and phenotypic correlation ( r ) between fresh weight in each combination of treatments by years and the CV.

Year	Treatment/Index	*h* ^2^	*r*
2019	W5	0.41	-0.12
	D10	0.23	-0.12
	D	0.23	-0.35***
2020	W10	0.64	-0.40***
	D10	0.33	-0.41***
	D	0.24	-0.42***
2021	W10	0.18	-0.44***
	D10	0.40	-0.45***
	D	0.43	-0.43***
	CV	0.35	

h^2^: genomic heritability, r: phenotypic correlation between fresh weight and coefficient of variation (CV), W5: watering for 5 d followed by no watering 5 d, W10: watering for 10 d followed by no watering 10 d, D10: no watering for 10 d followed by watering 10 d, D: no watering treatment, ***: significant at p< 0.001.

### 3.2 Model selection for random regression model

We evaluated the goodness-of-fit of the RRMs using NDVI and NDRE values for each treatment in 2021. Under all treatments, the best model was based on NDVI values using linear Legendre polynomials, that is, the order of nr was 1 (**Table 2**). The model with nr=0 exhibited the best NDRE values under all treatments (**Table S4**). This result indicates that only the intercept varied among genotypes in the RRM of NDRE values. Therefore, we employed the RRM of NDVI using nr=1 in later analyses. We built each RRM (Equation 1) using the time-series NDVI data for 2019, 2020, and 2021. We then calculated the genetic effect for the intercept and 1st coefficients of the Legendre polynomials (L0 and L1) for each year.

**Table 2:** The goodness-of-fit of random regression models (RRMs) with the normalized difference vegetation index (NDVI) values in 2021.

Treatment	*nr*	Loglik	AIC	*p*
W10	0	527.2572655	-1038.514531	8
	**1**	**739.1732966**	**-1458.346593**	**10**
	2	459.0197275	-892.0394551	13
D10	0	409.6136183	-803.2272366	8
	**1**	**553.0469996**	**-1086.093999**	**10**
	2	256.6404682	-487.2809363	13
D	0	704.2429918	-1392.485984	8
	**1**	**725.6443146**	**-1431.288629**	**10**
	2	458.2977954	-890.5955909	13

nr, the order of Legendre polynomial for the genetic effect; Loglik, log likelihood; AIC, Akaike’s information criterion; p, the number of parameters; W10, watering for 10 d followed by no watering 10 d; D10, no watering for 10 d followed by watering 10 d; D, no watering treatment. The best model in each treatment is bolded based on AIC.

### 3.3 Genetic correlation and genomic heritability of secondary traits

We estimated the genetic correlations between CV and L0 and CV and L1, and estimated the genomic heritability of L0, L1, and CV. In seven out of nine combinations of treatments by years, negative genetic correlations between CV and L0 or CV and L1 were observed (**Table 3**). L0 and L1 values for each genotype were associated with the intercepts and slopes of the time-series NDVI values. Therefore, these negative genetic correlations indicate that a low CV is associated with large intercepts and slopes of the time-series NDVI values. In 2019, there were no obvious patterns in genetic correlations or genomic heritability among the treatments (**Table 3**). However, in 2020, treatments W10 and D10 showed higher genetic correlations and genomic heritability than Treatment D for all the traits. In 2021, the genetic correlation of L0 was -0.2 (*p*< 0.01) in Treatment D, whereas W10 and D10 showed higher genetic correlations of -0.4 (*p*< 0.001) and -0.51 (p< 0.001), respectively.

**Table 3:** Genetic correlation between each parameter calculated in random regression models (RRMs) and coefficient of variation, and genomic heritability of genetic random regression coefficient of RRMs.

Year	Trait	Index	W5	W10	D10	D
2019	L0	*r_g_*	-0.26***		-0.03	-0.16*
		*h* ^2^	0.59		0.33	0.1
	L1	*r_g_*	0.08		-0.03	-0.17*
		*h* ^2^	0.79		0.54	0.31
2020	L0	*r_g_*		-0.32***	-0.4***	-0.15
		*h* ^2^		0.41	0.29	0.21
	L1	*r_g_*		-0.22**	-0.33***	-0.03
		*h* ^2^		0.79	0.43	0.1
2021	L0	*r_g_*		-0.4***	-0.51***	-0.2**
		*h* ^2^		0.11	0.39	0.38
	L1	*r_g_*		-0.35***	-0.31***	-0.34***
		*h* ^2^		0.7	0.67	0.29

L0: genetic effect for the intercept of Legendre polynomials in RRM, L1: the genetic effect for the 1st coefficient of Legendre polynomials in RRM, r_g_ genetic correlation, h^2^: genomic heritability, W5: watering for 5 d followed by no watering 5 d, W10: watering for 10 d followed by no watering 10 d, D10: no watering for 10 d followed by watering 10 d, D: no watering treatment, *, **, ***: significant at p< 0.05, 0.01, and, 0.001, respectively.

### 3.4 Case 1: within each combination of treatments by years

**Figure 6:**
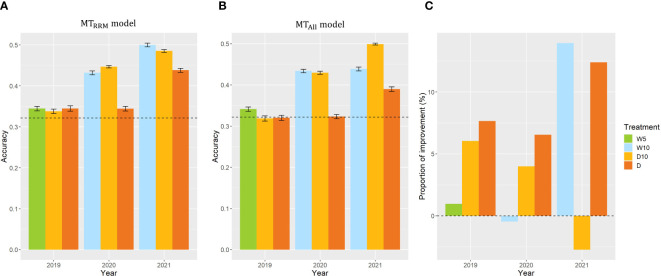
Prediction accuracy of ${\text{MT}}_{\text{RRM}}\;\text{model}$ and ${\text{MT}}_{\text{All}}\;\text{model}$ , and the comparison of ${\text{MT}}_{\text{RRM}}\;\text{model}$ and ${\text{MT}}_{\text{All}}\;\text{model}$ in Case1. **(A, B)** Prediction accuracy of ${\text{MT}}_{\text{RRM}}\;\text{model}$ and ${\text{MT}}_{\text{All}}\;\text{model}$ within each combination of treatments by years. Error bars represent standard error over 10 replicate cross-validations. A dashed line represents the prediction accuracy of the simple genomic prediction model. **(C)** The proportion of improvement calculated using the prediction accuracy of ${\text{MT}}_{\text{RRM}}\;\text{model}$ and ${\text{MT}}_{\text{All}}\;\text{model}$ . W5: watering for 5 d followed by no watering 5 d, W10: watering for 10 d followed by no watering 10 d, D10: no watering for 10 d followed by watering 10 d, D: no watering treatment.

In Case1, we used CV, which was calculated from the fresh weight collected in nine combinations of treatments by years, as the same target trait and built ${\text{MT}}_{\text{RRM}}\;\text{model}$ and ${\text{MT}}_{\text{All}}\;\text{model}$ in each combination of treatments by years. The prediction accuracy of the simple genomic prediction model was 0.32. The prediction accuracy of ${\text{MT}}_{\text{RRM}}\;\text{model}$ was higher than that of the simple genomic prediction model for all year treatments (**Figure 6A**). Three out of nine ${\text{MT}}_{\text{All}}\;\text{models}$ evaluating plant’s stability under drought. We focused on the ${\text{MT}}_{\text{RRM}}\;\text{model}$ . In 2019, there was no difference in the prediction accuracy among the treatments in ${\text{MT}}_{\text{RRM}}\;\text{model}$ . However, the prediction accuracies of treatments W10 and D10 in 2020 were 25% and 30% higher, respectively than those of Treatment D in 2020. In addition, the prediction accuracies of treatments W10 and D10 in 2021 were higher by 14% and 11%, respectively than those of Treatment D in 2021. These results indicate that the time-series MS data collected during the treatment, which changed the irrigation pattern, were more useful than those in the continuous drought treatment for predicting the CV in 2020 and 2021. To evaluate the importance of using RRMs, we compared ${\text{MT}}_{\text{RRM}}\;\text{model}$ and ${\text{MT}}_{\text{All}}\;\text{model}$ . In the seven combinations of treatments by years, the prediction accuracies of ${\text{MT}}_{\text{RRM}}\;\text{model}$ were higher than those of ${\text{MT}}_{\text{All}}\;\text{model}$ (**Figure 6C**). In particular, the proportion of improvement was 14% for Treatment W10 in 2021.

### 3.5 Case 2: using a small dataset of secondary traits

**Figure 7:**
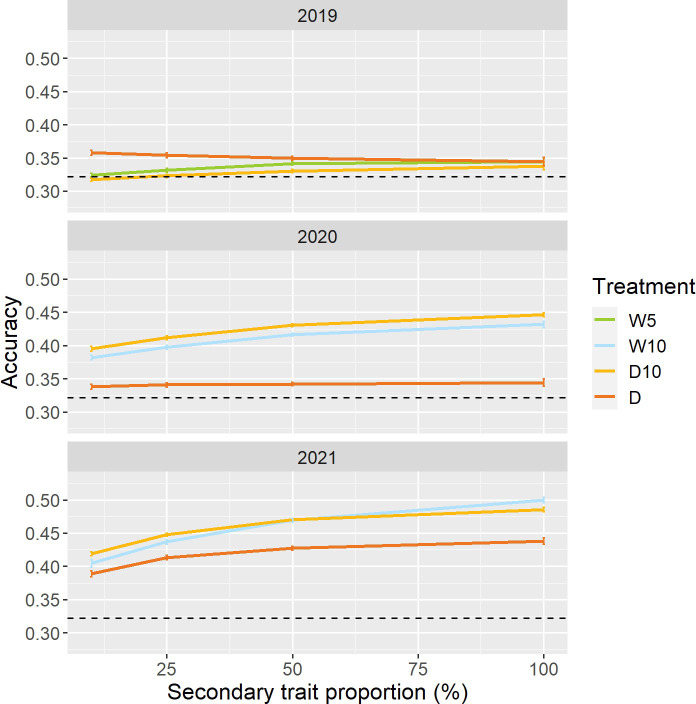
Prediction accuracy of ${\text{MT}}_{\text{RRM}}\;\text{model}$ when a specific proportion of genotypes in the training set did not have secondary trait data. Dashed line represents the prediction accuracy of simple genomic prediction model. Error bars represent standard error over 50 replicate cross-validations. W5: watering for 5 d followed by no watering 5 d, W10: watering for 10 d followed by no watering 10 d, D10: no watering for 10 d followed by watering 10 d, D: no watering treatment.

To reduce the cost and labor required to create the prediction model, we changed the proportion of genotypes collected as secondary trait data in the training set. In 2020 and 2021, except for Treatment D in 2020, the prediction accuracy for all combinations of treatments by years increased as the proportion of genotypes with secondary trait data increased (**Figure 7**). In Treatment D10 of 2020 and 2021, even when the proportions of data with secondary traits were 10%, the prediction accuracies of ${\text{MT}}_{\text{RRM}}\;\text{models}$ were 0.40 and 0.42. These prediction accuracies were 23% and 30% higher than those of the simple genomic prediction model, respectively. Moreover, there was no obvious pattern in the proportion of improvement (**Figure S3**).

### 3.6 Case 3: across years for the same type of treatment

**Figure 8:**
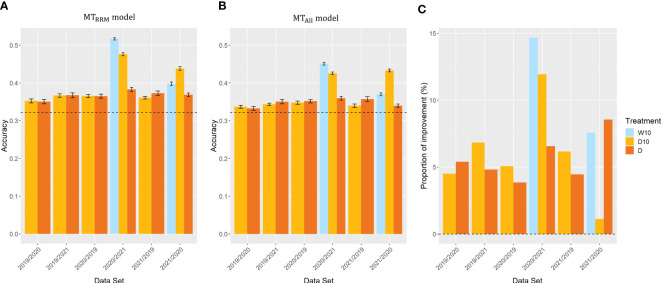
Prediction accuracy of ${\text{MT}}_{\text{RRM}}\;\text{model}$ and ${\text{MT}}_{\text{All}}\;\text{model}$ , and the proportion of improvement in Case3. Item names, such as 2019/2020, represent the training year and test year. **(A, B)** Prediction accuracy of $rmMT_{RRM}\;model$ and ${\text{MT}}_{\text{All}}\;\text{model}$ among years in the same treatment. Dashed line represents the prediction accuracy of simple genomic prediction model. Error bars represent standard error over 10 replicate cross-validations. **(C)** The proportion of improvement in each combination. W5: watering for 5 d followed by no watering 5 d, W10: watering for 10 d followed by no watering 10 d, D10: no watering for 10 d followed by watering 10 d, D: no watering treatment.

In Case3, we evaluated the across-year predictions for each treatment. In all three treatments, ${\text{MT}}_{\text{RRM}}\;\text{models}$ and ${\text{MT}}_{\text{All}}\;\text{models}$ outperformed the simple genomic prediction model (**Figures 8A, B**). In cross-validations with the 2020 and 2021 datasets, the prediction accuracy of ${\text{MT}}_{\text{RRM}}\;\text{models}$ for Treatments W10 and D10 were on average 42% and 42% higher than that of the simple genomic prediction model, respectively. The proportion of improvement in all ${\text{MT}}_{\text{RRM}}\;\text{models}$ was greater than zero (**Figure 8C**). The proportion of improvement was, on average, 11, 6, and 6% for treatments W10, D10, and D, respectively. This result indicates that the time-series MS data should be modeled using RRM for across-year predictions.

## 4 Discussion

### 4.1 Usefulness of MS data for predicting plant’s stability under drought stress

In this study, we compared the prediction accuracy of a simple genomic prediction model and a genomic prediction model using time-series NDVI values as secondary traits to predict the CV (**Figures 6**–**8**). In soybeans, a relationship between NDVI and drought tolerance has been reported (Zhou et al., 2020). Except for Case2 in 2019, all genomic prediction models using time-series NDVI values as a secondary trait showed higher prediction accuracy than a simple genomic prediction model (**Figures 6**–**8**). These results suggest that time-series MS data are useful for predicting the CV.

In 2020 and 2021, the prediction accuracy of treatments W10 and D10, which changed irrigation treatments, was higher than that of Treatment D, which was the no watering treatment, in the same year (**Figures 6**, **7**). A relationship between plant responses to changes in irrigation and drought tolerance has been reported. In soybeans, slow wilting resulted in drought tolerance (Fletcher et al., 2007; Pathan et al., 2014; Valliyodan et al., 2017; Ye et al., 2020) and a relationship between slow wilting and NDVI values under drought is observed (Zhou et al., 2020). Additionally, the speed of recovery from drought stress is associated with drought tolerance in several crop species (Hayano-Kanashiro et al., 2009; Kränzlein et al., 2021), including soybean (Hossain et al., 2014). However, no study has evaluated the plant’s stability under drought stress using time-series NDVI changes caused by changes in irrigation. This result indicates that plant responses to irrigation changes are useful for evaluating plant’s stability under drought stress.

In this study, the overall prediction accuracy of CV was lower in 2019 than that in the other years. There are two possible reasons for this result. One possible reason is that NDVI was measured in 2019 (four times) less frequently than in 2020 (six times) and 2021 (seven times). The small number of measurements may not capture the time-series changes in NDVI well. In addition, we collected NDVI values on the first day after the irrigation change in Treatment D10 in 2019 (**Figure 3**). The second possible reason is the weather conditions. In 2020 and 2021, the soil moisture content increased during the irrigation period and decreased during the non-irrigation period (**Figure S1**). In contrast, in 2019, treatments W5 and D10 showed the opposite trend of increasing soil moisture content during the last non-irrigation period. In 2019, it rained for 6 out of 10 days from 25 August to 3 September during the period of no irrigation in Treatment D10. The lack of drought stress during this period might have resulted in a failure to capture the plant response to irrigation change and low prediction accuracy in 2019.

### 4.2 The advantage of using random regression

In Case1, MTRRM model, which modeled time-series NDVI data and used L0 and L1 (genetic random regression coefficients) as secondary traits in the MTM, generally showed a higher prediction accuracy than MTAll model, which treated time-series NDVI data as independent traits and used each day’s NDVI value as a secondary trait in the MTM (**Figure 6**). It was reported that the RRM is superior to the MTM in modeling time-series data (Kranis et al., 2007; Mota et al., 2013). In addition, there are problems with over-parameterization and high computational requirements when treating time-series data as independent traits and constructing MTMs (Misztal et al., 2000; Oh and See, 2008; Moreira et al., 2020). In terms of calculation efficiency, RRM is useful for modeling time-series data.

Campbell et al. (Campbell et al., 2018) built RRM using time-series projected shoot area (PSA) data for 179 rice lines obtained in their own study. Using the built-in RRM (Campbell et al., 2018), Campbell predicted time-series changes in PSA for new 178 rice lines collected in a different year (Campbell et al., 2017). This result suggests that RRM is useful for predictions across years. In our study, MTRRM model is superior to MTAll model in terms of prediction across years in Case3 (**Figure 8**). MTRRM model can build a model using all-day data of NDVI value in each year, whereas MTAll model can only use dates that are common across all years. This difference in the amount of data may be the reason for the difference in prediction accuracy. When using MTAll model for predictions across years, it is necessary to match the data measurement dates. It was difficult to collect data on a desired date because of weather conditions or equipment problems. At this point, the RRM can be built without considering the measurement date and number of days of data measurement.

### 4.3 Utility for breeding

Environmental stability, also, the stability of phenotypes over environments, is an important target in breeding (Tollenaar and Lee, 2002; Sabaghnia et al., 2008; Mickelbart et al., 2015; Blum, 2018). The CV of phenotypic values over environments can be a good indicator for selecting stable genotypes. To evaluate genotypes’ stability across environments, it is necessary to conduct multi-environmental experiments which require high costs and labor (Fan et al., 2007; Karimizadeh et al., 2013; Akter et al., 2015; Lal et al., 2022). In this study, we proposed a method to measure the response to changes in irrigation by remote sensing and model it with RRM at a single location in multiple years. We found that genetic variation captured by the RRM was associated with plant’s stability under drought stress, even with only one location in three years. The results of Case1 validation suggest that genetic variation captured by measuring and modeling plant responses to irrigation in a single environment changes can predict the stability over nine environments (**Figure 6**). The results of Case2 validation indicate that even when 10% of genotypes in a training set had secondary trait data, MTRRM model was more accurate than a simple genomic prediction model in treatments W10 and D10 in 2020 and 2021 (**Figure 7**). Therefore, it is possible to reduce the number of genotypes measured for secondary traits in the training set. The results of Case3 validation also suggest that we can predict the plant’s stability across years (**Figure 8**). Once a prediction model is constructed, the stability of unknown genotypes can be predicted based on time-series NDVI data. The results of Case2 and Case3 validations also demonstrate the flexibility of MTRRM model. When the stability of unknown genotypes under drought is evaluated in a single environment, it greatly reduces the time and cost, and thus streamlines breeding schemes. When calculating CVs, experimental designs with repetitions per treatment and year combination are usually used (Mohammadi and Amri, 2008; Das et al., 2010; Küchenmeister et al., 2012; Di Matteo et al., 2016). ¨ In this study, priority was given to increasing the number of genotypes over replications. This is because it is known that maximizing the number of genotypes (even with one replication) is the most effective when the number of plots is limited for QTL analysis and genomic prediction (Knapp and Bridges, 1990; Lorenz, 2013). Given the lack of replications and the small size of microplots, the fresh weight is probably influenced by non-genetic factors. However, the prediction accuracy of CV was improved by using information obtained from the methods for measuring and modeling changes in time-series NDVI data. Therefore, the effectiveness of the developed method was confirmed. In order to apply these measuring and modeling methods in breeding selection, it is necessary to associate the information obtained from the developed methods with drought tolerance-related traits and even yield-related traits in expanded field trials.

In this study, we developed a method to measure and model plant responses to irrigation changes and confirmed their association with plant’s stability under drought stress. Drone-based HTP allows us to capture time-series plant responses to environmental and irrigation changes (Chen et al., 2014; Dodig et al., 2021), and measuring and modeling plant responses to these environmental changes will provide important insights that have not yet been obtained previously (Arnold et al., 2019; Moreira et al., 2020). This developed method will contribute to the study of abiotic stress and genetic improvement of soybean.

## Data availability statement

The original contributions presented in the study are included in the article/**Supplementary Material**. Further inquiries can be directed to the corresponding author.

## Author contributions

KS: Methodology, software, formal analysis, data curation, writing—original draft preparation and visualization. YT: investigation, data curation and project administration. KM: methodology and software. HI: conceptualization, investigation, data curation, writing—review and editing, supervision, project administration and funding acquisition. AK: Conceptualization, resources, data curation, and funding acquisition. YO, YY, HirT, HidT, MT, HTs, MN, and TF: Conceptualization, data curation, and funding acquisition. All authors contributed to the article and approved the submitted version.
