# Supplementary material for: Random regression for modeling soybean plant response to irrigation changes using time-series multispectral data
Source: Front Plant Sci. 2023 Jul 5;14:1201806. doi: 10.3389/fpls.2023.1201806 (PMC10354427; doi:10.3389/fpls.2023.1201806)
Supplement: Supplementary file 1 [file Image_1.pdf]

(a)

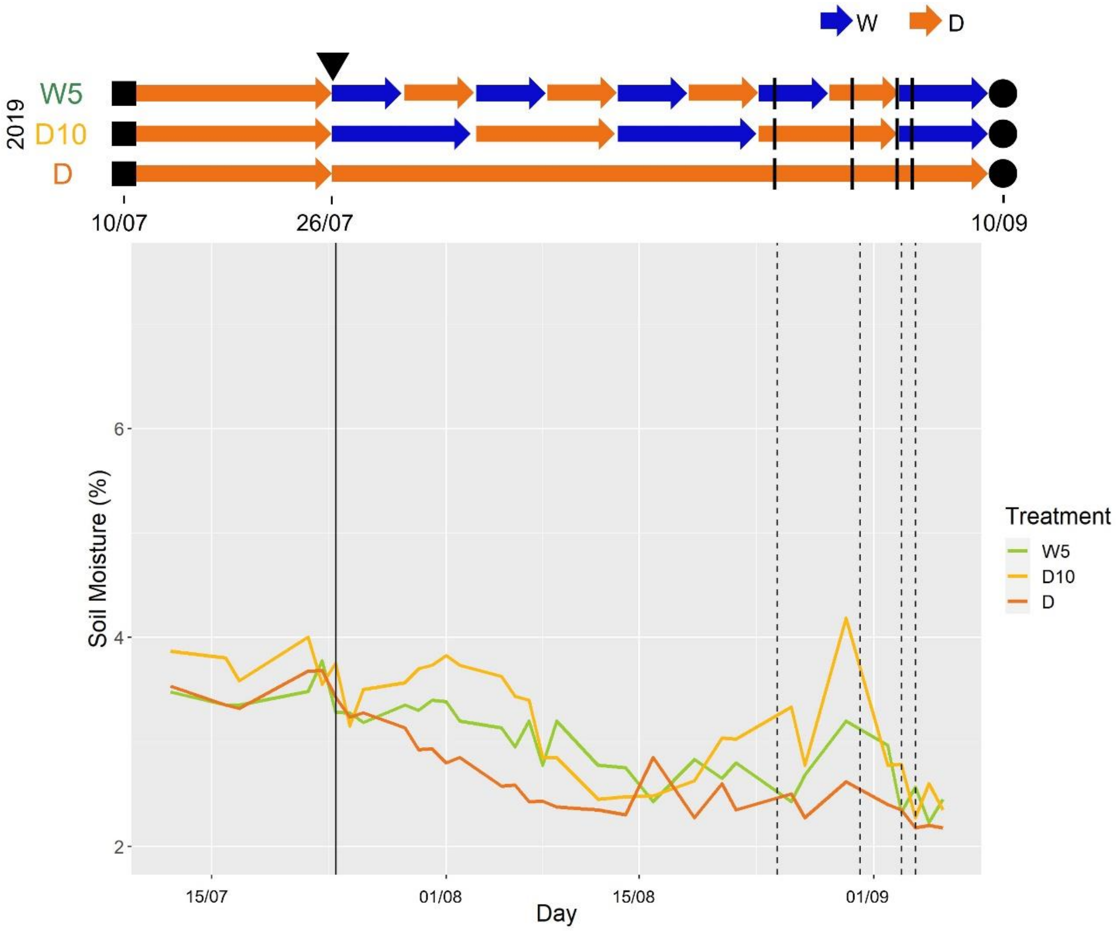

Figure S1. The cycle of irrigation treatment and the day change of soil moisture content in each combination of treatments by years. W5: watering for 5 d followed by no watering 5 d, W10: watering for 10 d followed by no watering 10 d, D10: no watering for 10 d followed by watering 10 d, D: no watering treatment. Two colors of arrows mean the irrigation treatment: not irrigated period (orange), irrigated period (blue). The solid line represents the date of thinning and dashed lines represent the date of UAV measurements. **(a)** in 2019, **(b)** in 2020, **(c)** in 2021.

(b)

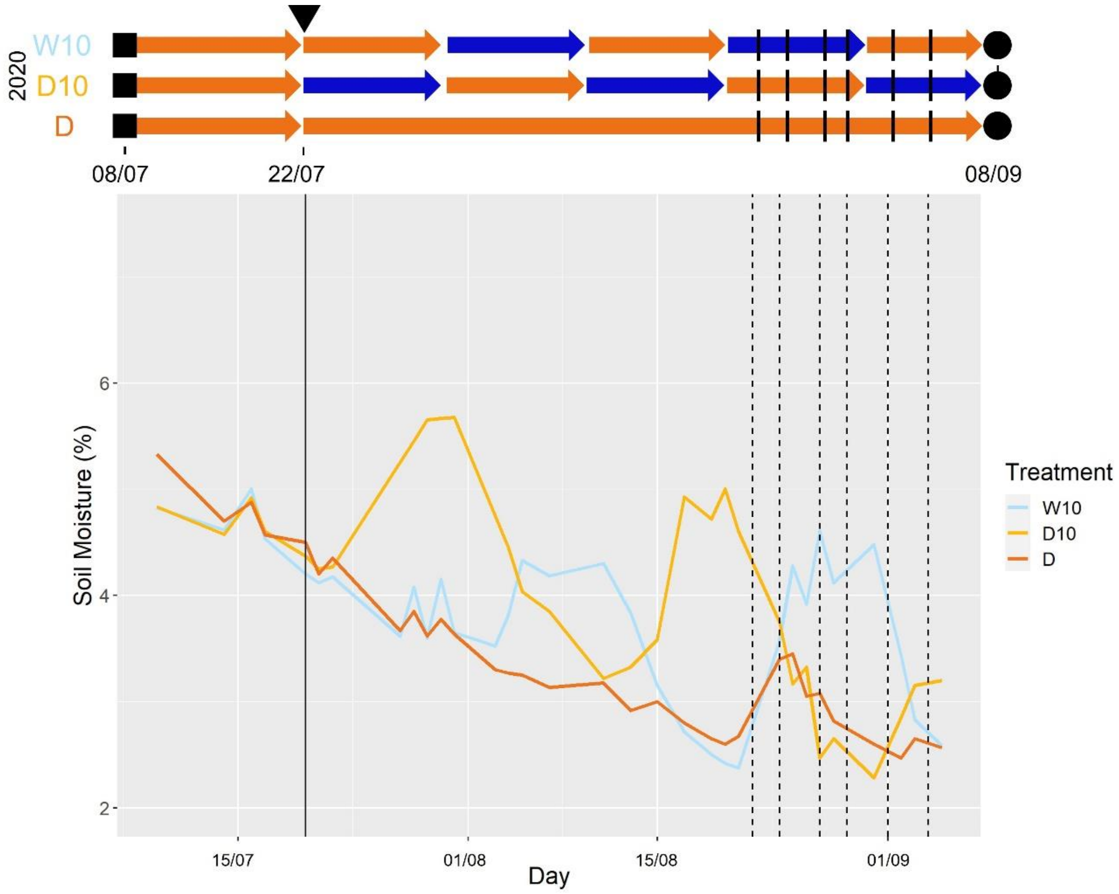

Figure S1. The cycle of irrigation treatment and the day change of soil moisture content in each combination of treatments by years. W5: watering for 5 d followed by no watering 5 d, W10: watering for 10 d followed by no watering 10 d, D10: no watering for 10 d followed by watering 10 d, D: no watering treatment. Two colors of arrows mean the irrigation treatment: not irrigated period (orange), irrigated period (blue). The solid line represents the date of thinning and dashed lines represent the date of UAV measurements. **(a)** in 2019, **(b)** in 2020, **(c)** in 2021.

(c)

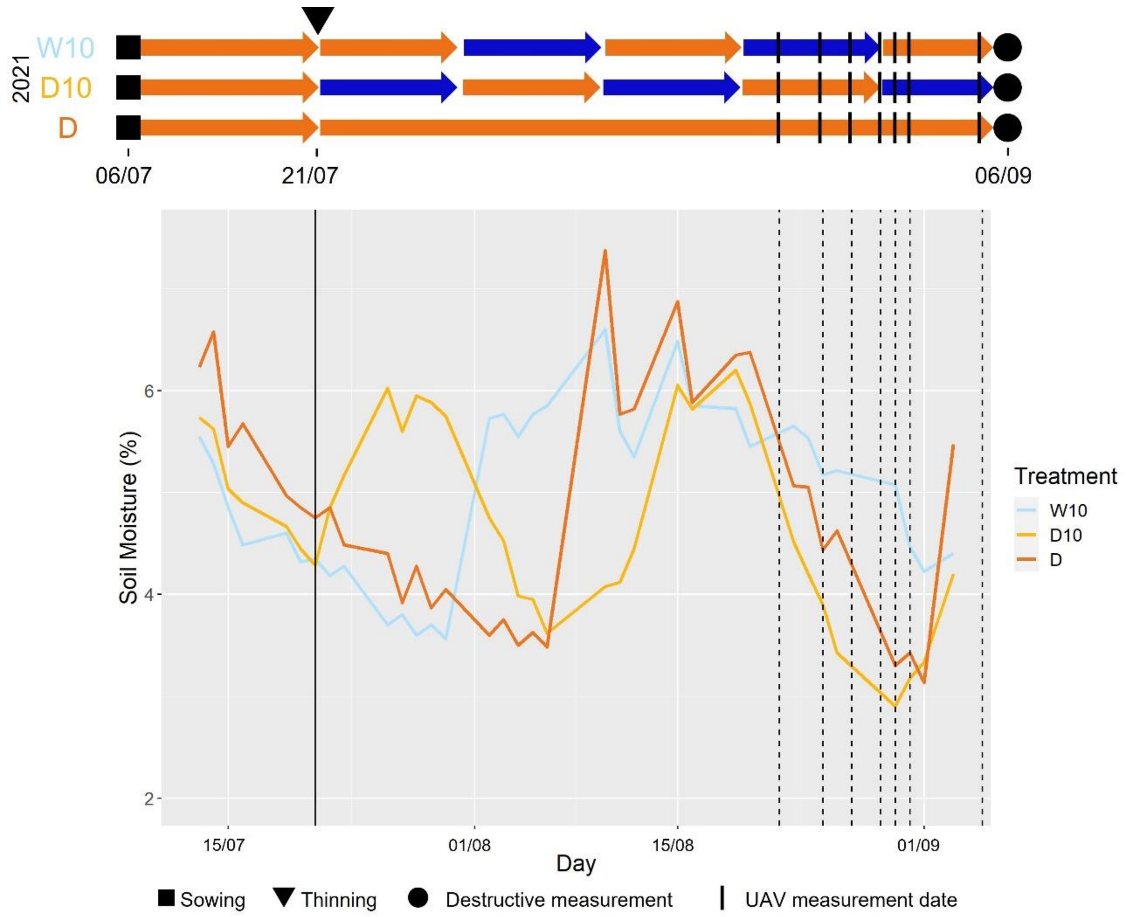

Figure S1. The cycle of irrigation treatment and the day change of soil moisture content in each combination of treatments by years. W5: watering for 5 d followed by no watering 5 d, W10: watering for 10 d followed by no watering 10 d, D10: no watering for 10 d followed by watering 10 d, D: no watering treatment. Two colors of arrows mean the irrigation treatment: not irrigated period (orange), irrigated period (blue). The solid line represents the date of thinning and dashed lines represent the date of UAV measurements. **(a)** in 2019, **(b)** in 2020, **(c)** in 2021.
