# Supplementary material for: Random regression for modeling soybean plant response to irrigation changes using time-series multispectral data
Source: Front Plant Sci. 2023 Jul 5;14:1201806. doi: 10.3389/fpls.2023.1201806 (PMC10354427; doi:10.3389/fpls.2023.1201806)
Supplement: Supplementary file 2 [file Image_2.pdf]

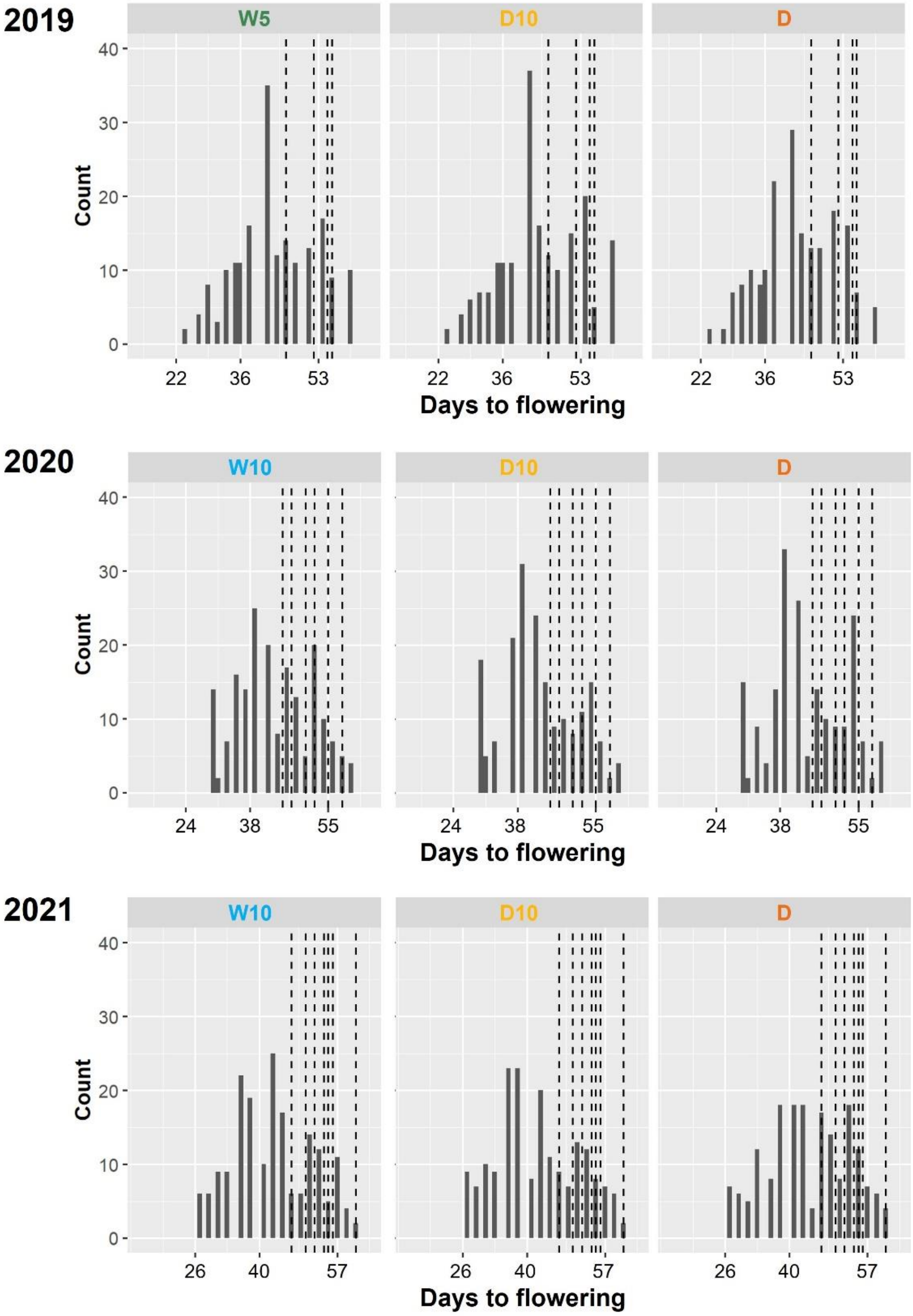

Figure S2. Histogram of days to flowering in each combination of treatments by years; W5: watering for 5 d followed by no watering 5 d, W10: watering for 10 d followed by no watering 10 d, D10: no watering for 10 d followed by watering 10 d, D: no watering treatment. Dashed lines represent the date of UAV measurements.
