# Supplementary material for: Random regression for modeling soybean plant response to irrigation changes using time-series multispectral data
Source: Front Plant Sci. 2023 Jul 5;14:1201806. doi: 10.3389/fpls.2023.1201806 (PMC10354427; doi:10.3389/fpls.2023.1201806)
Supplement: Supplementary file 4 [file Table_1.docx]

**Table S1 The description of 178 accessions that are used in this study. We used soybean genetic resources, registered as the mini core collections in the National Institute of Agrobiological Sciences (NIAS) gene bank.**

| 5002T | C1329 | GmJMC003 | GmJMC004 | GmJMC007 |
| --- | --- | --- | --- | --- |
| GmJMC009 | GmJMC013 | GmJMC016 | GmJMC017 | GmJMC021 |
| GmJMC023 | GmJMC025 | GmJMC026 | GmJMC028 | GmJMC030 |
| GmJMC031 | GmJMC032 | GmJMC033 | GmJMC034 | GmJMC037 |
| GmJMC039 | GmJMC040 | GmJMC041 | GmJMC044 | GmJMC047 |
| GmJMC049 | GmJMC050 | GmJMC051 | GmJMC053 | GmJMC054 |
| GmJMC055 | GmJMC056 | GmJMC057 | GmJMC058 | GmJMC059 |
| GmJMC060 | GmJMC061 | GmJMC062 | GmJMC063 | GmJMC064 |
| GmJMC065 | GmJMC067 | GmJMC068 | GmJMC069 | GmJMC076 |
| GmJMC077 | GmJMC078 | GmJMC079 | GmJMC080 | GmJMC082 |
| GmJMC085 | GmJMC091 | GmJMC092 | GmJMC093 | GmJMC095 |
| GmJMC096 | GmJMC097 | GmJMC098 | GmJMC099 | GmJMC100 |
| GmJMC101 | GmJMC102 | GmJMC104 | GmJMC105 | GmJMC106 |
| GmJMC110 | GmJMC111 | GmJMC112 | GmJMC114 | GmJMC116 |
| GmJMC117 | GmJMC121 | GmJMC126 | GmJMC128 | GmJMC130 |
| GmJMC131 | GmJMC133 | GmJMC137 | GmJMC139 | GmJMC145 |
| GmJMC149 | GmJMC158 | GmJMC161 | GmJMC167 | GmJMC172 |
| GmJMC177 | GmJMC179 | GmJMC180 | GmJMC184 | GmWMC001 |
| GmWMC006 | GmWMC010 | GmWMC011 | GmWMC012 | GmWMC014 |
| GmWMC015 | GmWMC018 | GmWMC022 | GmWMC024 | GmWMC027 |
| GmWMC029 | GmWMC035 | GmWMC036 | GmWMC038 | GmWMC042 |
| GmWMC045 | GmWMC046 | GmWMC048 | GmWMC066 | GmWMC070 |
| GmWMC071 | GmWMC072 | GmWMC073 | GmWMC074 | GmWMC075 |
| GmWMC083 | GmWMC084 | GmWMC086 | GmWMC089 | GmWMC094 |
| GmWMC103 | GmWMC107 | GmWMC108 | GmWMC109 | GmWMC115 |
| GmWMC118 | GmWMC119 | GmWMC120 | GmWMC122 | GmWMC123 |
| GmWMC124 | GmWMC125 | GmWMC127 | GmWMC129 | GmWMC132 |
| GmWMC134 | GmWMC135 | GmWMC136 | GmWMC140 | GmWMC141 |
| GmWMC142 | GmWMC143 | GmWMC144 | GmWMC146 | GmWMC147 |
| GmWMC148 | GmWMC151 | GmWMC152 | GmWMC153 | GmWMC154 |
| GmWMC155 | GmWMC156 | GmWMC159 | GmWMC160 | GmWMC162 |
| GmWMC163 | GmWMC164 | GmWMC165 | GmWMC166 | GmWMC168 |
| GmWMC169 | GmWMC171 | GmWMC173 | GmWMC174 | GmWMC175 |
| GmWMC176 | GmWMC178 | GmWMC181 | GmWMC182 | GmWMC183 |
| GmWMC185 | GmWMC186 | GmWMC188 | GmWMC189 | GmWMC190 |
| GmWMC191 | GmWMC192 | Houjaku Kuwazu |  |  |
