# Supplementary material for: Random regression for modeling soybean plant response to irrigation changes using time-series multispectral data
Source: Front Plant Sci. 2023 Jul 5;14:1201806. doi: 10.3389/fpls.2023.1201806 (PMC10354427; doi:10.3389/fpls.2023.1201806)
Supplement: Supplementary file 5 [file Table_2.docx]

**Table S2** **The equations of two vegetation indices (VIs) which are used in this study.**

| **Index** | **Equation** |
| --- | --- |
| Normalized difference vegetation index (NDVI) | $(\rho NIR-\rho RED)/$  $(\rho NIR+\rho RED)$ |
| Normalized difference red-edge index (NDRE) | $(\rho NIR-\rho RE)/$  $(\rho NIR+\rho RE)$ |

Note: $\rho RED, \rho RE, \rho NIR$ represent the spectral reflectance of red (660 nm or 650 nm), red-edge (725 nm or 730 nm), and near-infrared (850 nm or 840 nm).
