# Supplementary material for: Random regression for modeling soybean plant response to irrigation changes using time-series multispectral data
Source: Front Plant Sci. 2023 Jul 5;14:1201806. doi: 10.3389/fpls.2023.1201806 (PMC10354427; doi:10.3389/fpls.2023.1201806)
Supplement: Supplementary file 6 [file Table_3.docx]

**Table S3** ﻿**The ratio of flowering at the timings of unmanned aerial vehicle measurements in each combination of treatments by years.**

|  |  |  | **The Ratio of Flowering** | | | |
| --- | --- | --- | --- | --- | --- | --- |
| **Year** | **Date** | **Days After Sowing** | **W5** | **W10** | **D10** | **D** |
| 2019 | 25/08 | 46 | 0.68 |  | 0.66 | 0.68 |
|  | 31/08 | 52 | 0.81 |  | 0.79 | 0.85 |
|  | 03/09 | 55 | 0.9 |  | 0.9 | 0.94 |
|  | 04/09 | 56 | 0.95 |  | 0.93 | 0.97 |
| 2020 | 22/08 | 45 |  | 0.57 | 0.65 | 0.57 |
|  | 24/08 | 47 |  | 0.66 | 0.7 | 0.64 |
|  | 27/08 | 50 |  | 0.75 | 0.79 | 0.74 |
|  | 29/08 | 52 |  | 0.86 | 0.85 | 0.79 |
|  | 01/09 | 55 |  | 0.91 | 0.93 | 0.92 |
|  | 04/09 | 58 |  | 0.98 | 0.98 | 0.96 |
| 2021 | 22/08 | 47 |  | 0.7 | 0.7 | 0.62 |
|  | 25/08 | 50 |  | 0.74 | 0.74 | 0.7 |
|  | 27/08 | 52 |  | 0.81 | 0.81 | 0.74 |
|  | 29/08 | 54 |  | 0.88 | 0.88 | 0.84 |
|  | 30/08 | 55 |  | 0.91 | 0.92 | 0.91 |
|  | 31/08 | 56 |  | 0.91 | 0.92 | 0.91 |
|  | 05/09 | 61 |  | 1 | 1 | 1 |

W5: watering for 5 d followed by no watering 5 d, W10: watering for 10 d followed by no watering 10 d, D10: no watering for 10 d followed by watering 10 d, D: no watering treatment.
