# Supplementary material for: Random regression for modeling soybean plant response to irrigation changes using time-series multispectral data
Source: Front Plant Sci. 2023 Jul 5;14:1201806. doi: 10.3389/fpls.2023.1201806 (PMC10354427; doi:10.3389/fpls.2023.1201806)
Supplement: Supplementary file 7 [file Table_4.docx]

**Table S4** ﻿**The goodness-of-fit of random regression models (RRMs) with the normalized difference red-edge (NDRE) values in 2021.**

| Treatment | $nr$ | Loglik | AIC | $p$ |
| --- | --- | --- | --- | --- |
| **W10** | **0** | **794.7129** | **-1573.43** | **8** |
|  | 1 | 731.8879 | -1443.78 | 10 |
|  | 2 | 425.5259 | -825.052 | 13 |
| **D10** | **0** | **598.1608** | **-1180.32** | **8** |
|  | 1 | 554.6491 | -1089.3 | 10 |
|  | 2 | 308.1588 | -590.318 | 13 |
| **D** | **0** | **696.3551** | **-1376.71** | **8** |
|  | 1 | 539.5232 | -1059.05 | 10 |
|  | 2 | 437.6705 | -849.341 | 13 |

$nr$: the order of Legendre polynomial for the genetic effect, Loglik: log likelihood, AIC: Akaike’s information criterion, $p$: the number of parameters, W10: watering for 10 d followed by no watering 10 d, D10: no watering for 10 d followed by watering 10 d, D: no watering treatment. The best model in each treatment is bolded based on AIC.
